# Growth and yield of maize in response to reduced fertilizer application and its impacts on population dynamics and community biodiversity of insects and soil microbes

**DOI:** 10.3389/fpls.2024.1362905

**Published:** 2024-05-24

**Authors:** Yan Zou, Likun Li, Yanhui Wang, Ruichuan Duan, Hejie Dong, Yuhan Zhang, Zhengze Du, Fajun Chen

**Affiliations:** ^1^ Department of Entomology, College of Plant Protection, Nanjing Agricultural University, Nanjing, China; ^2^ College of Life Sciences, Nanjing Agricultural University, Nanjing, China

**Keywords:** maize, fertilizer reduction, biomass and yield, insects, soil microbes

## Abstract

In the North China Plain, farmers are using excessive amounts of fertilizer for the production of high-yield crop yield, which indirectly causes pollution in agricultural production. To investigate an optimal rate of fertilizer application for summer maize, the fertilizer reduction experiments with 600 kg/ha NPK (N: P_2_O_5_: K_2_O = 28: 8: 10) as normal fertilizer application (NFA), (i.e., 100F), were conducted successively during 2020 and 2021 to study the effects of reduced fertilizer rates, including 90% (540 kg/ha; i.e., 90F), 80% (480 kg/ha; i.e., 80F), 62.5% (375 kg/ha; i.e., 62.5F) and 50% (300 kg/ha; i.e., 50F) of NFA, on the plant growth of maize, the dynamics of key population abundances and community diversity of insects, and the composition and diversity of microbial community and finally to find out the N-metabolic enzymes’ activity in soil. Our findings revealed that the fertilizer reduction rates by 10% - 20% compared to the current 100% NFA, and it has not significantly affected the plant growth of maize, not only plant growth indexes but also foliar contents of nutrients, secondary metabolites, and N-metabolic enzymes’ activity. Further, there was no significant alteration of the key population dynamics of the Asian corn borer (*Ostrinia furnacalis*) and the community diversity of insects on maize plants. It is interesting to note that the level of N-metabolic enzymes’ activity and microbial community diversity in soil were also not affected. While the fertilizer reduction rate by 50% unequivocally reduced field corn yield compared to 100% NFA, significantly decreased the yield by 17.10%. The optimal fertilizer application was calculated as 547 kg/ha (i.e., 91.17% NFA) based on the simulation analysis of maize yields among the five fertilizer application treatments, and the fertilizer application reduced down to 486 kg/ha (i.e., 81.00% NFA) with a significant reduction of maize yield. These results indicated that reduced the fertilizer application by 8.83% - 19.00% is safe and feasible to mitigate pollution and promote sustainable production of maize crops in the region.

## 1 Introduction

Maize (*Zea mays* L.) is a crucial crop for global food security, and China is the worldly 2nd-largest producer of maize crop with approximately 43.55 million hectares of cultivated land, accounting for 36.8% of the country’s grain acreage and 39.9% of its production (Manevski et al., 2016; Bai et al., 2021; Nbs, 2021). While maize is the primary summer crop in northern China, and it faces challenges such as excessive nitrogen and phosphorus input, low yield, water waste, and environmental pollution etc (Bellarby et al., 2017; Wang et al., 2022d).

Maize yield in the North China Plain was around 8054.8 kg/ha in 2018, which exceeds the national average of 6104.3 kg/ha (Nbs, 2021) but falls beneath that in developed countries, such as, the United States (11,075 kg ha ^-1^), France (8,821 kg ha^-1^) and Germany (8,139 kg/ha) during the same year (Nbs, 2021; Fao, 2022; Nbs, 2022; Yu et al., 2023). It is also much lower than the theoretical maize yield potential of 17600 kg/ha^-1^ in the North China Plain (Meng et al., 2013). A range of factors, including variety, field management practices, water availability, light, and heat etc., can affect crop yield. However, fertilizer is often considered the most readily available means of boosting yields and increasing profits for farmers. Over the years, the total amount of fertilizer used has increased substantially from 0.07 MMT in 1950 to 49.6 MMT in 2010 in China. In fact, a significant portion of this increase (74.7%) was due to changes in fertilizer application rates. Since the 1980s, corn, vegetables, and fruits have been the primary crops driving growth in fertilizer consumption. These three crops accounted for 90.6% of the overall increase in fertilizer usage during the 2000s (Chen et al., 2018).

Maize has the highest fertilizer application rate among grain-crops. This is due in part to its higher nutrient uptake capacity, longer growing period, and relatively higher yield. In addition, many smallholders in China are willing to invest more in their farmland despite being aware of having invested too much in fertilizers, partly due to seeking a lower-risk income. However, many studies show that fertilizer inputs in northern China are excessive (Ju et al., 2007; Xiao et al., 2021; Zhang et al., 2022; Yu et al., 2023). Over-usage of chemical fertilizers has caused serious environmental problems, including surface water eutrophication, groundwater pollution, soil degradation, and greenhouse gas emissions from excess nitrogen that is either lost by immersion into water or volatilized into air as ammonia and N_2_O etc (Yan et al., 2013; Norse and Ju, 2015; Zhou et al., 2018; Yang et al., 2022). It has shown that excessive fertilizer usage may not necessarily increase yields and can even reduce total revenue to some extent (Abdo et al., 2022; Wang et al., 2022d). In fact, current agricultural fertilization practices, which involve applying 550–600 kg nitrogen fertilizer per hectare and per growing season, do not significantly improve crop yields, even result in a twofold loss of nitrogen to the environment, and optimal fertilizer application should be reduced by 30%–60% to achieve better yields and minimize negative environmental impacts (Ju et al., 2009).

The excessive use of chemical fertilizers not only leads to resource waste but is also harmful to the farmland. We carried out a series of fertilizer reduction experiments with 600 kg/ha NPK fertilizer as normal application usage in the region of Jiyang districts, Jinan City, Shandong Province of China. The plant growth and yield of maize and the following impacts on key population dynamics of insect pests, and community biodiversity of insects and soil microbes were measured. This study aims to clarify the effects of fertilizers reduction on field ecology and crop yield. The objectives of this study further aims to provide practical guidance of the optimal fertilizer application for local agricultural production, and simultaneously minimize the negative environmental impacts of excessive fertilizer usage.

## 2 Material and methods

### 2.1 Experimental site description

**Figure 1:**
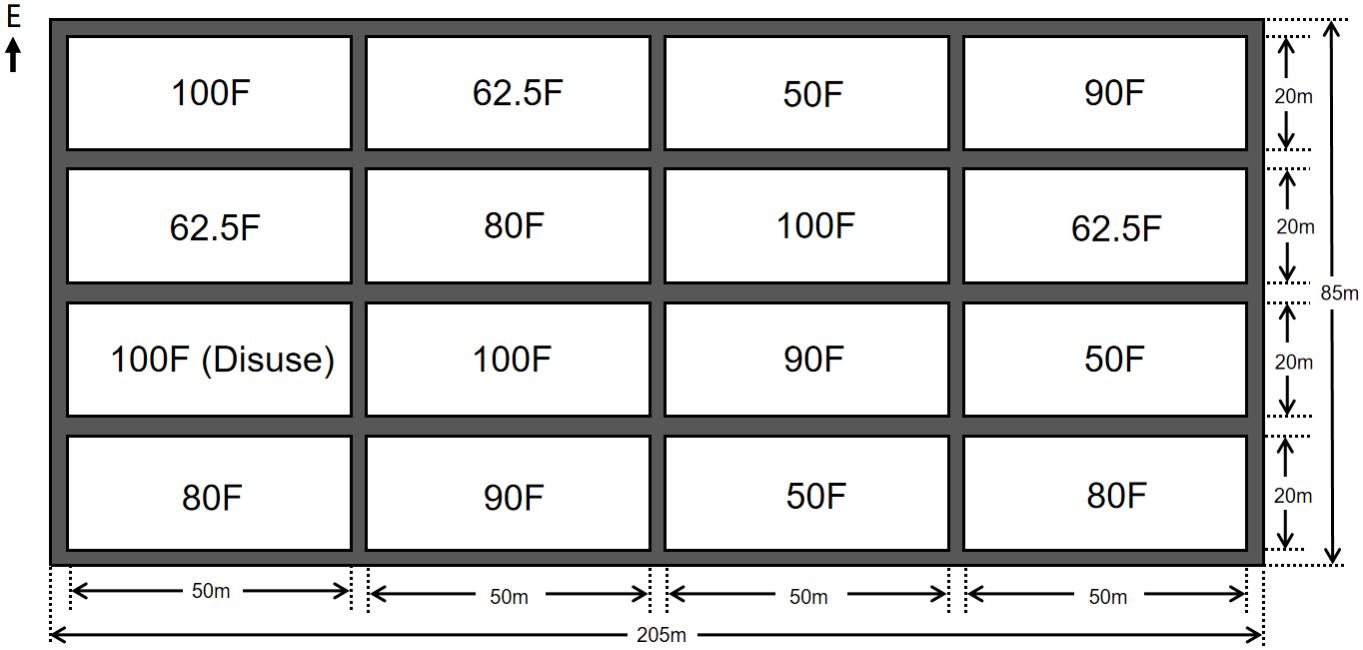
Field layout model of the fertilizer application treatments to affect the plant growth and yield production of maize, and dynamics of key populations and community diversity of insects. (100F - normal fertilizer application (NFA) of 600 kg NPK (N: P_2_O_5_: K_2_O = 28: 8: 10) per hectare (ha.); 90F, 80F, 62.5F and 50F - the four fertilizer reduction gradients with 90% (540kg/ha), 80% (480kg/ha), 62.5% (375kg/ha) and 50% (300kg/ha) of NFA, respectively; The row spacing and plant distance of corn was 0.80 m and 0.20 m respectively. The same in the following figures).

The experimental field was located at Nanxin Village (117.10806S; 36.88213N), Jiyang district, Jinan City, Shandong Province of China. It has a rectangular shape and covers an area of about 1.75 hectares, measuring 205 meters from north to south, and 85 meters from east to west (seen in **Figure 1**). The topography of the experimental field is typical of the North China Plain. The soil type is fluvo-aquic, and it has an average annual temperature of 12.8°C, a frost-free period of 195 days, solar radiation of 124.4 kcal/cm^2^, and precipitation of 583.3 mm. The basic fertility parameters of soil were as follows: total carbon, 6.70 g/kg; total nitrogen, 0.76 g/kg; available phosphorus, 28.97 mg/kg; available potassium, 99.84 mg/kg; and organic carbon, 6.63 g/kg. The crops are planted in a rotation system, with maize grown from June to October annually, and wheat grown from October to June in the following seasons. After harvest, the straws of maize and wheat were pulverized and uniformly returned to the soil. The planting methods used were consistent with those of local farmers.

### 2.2 Crop planting and sampling collection

The maize variety “Qihua 703” was selected and bought from Beijing Lantron Seed Co., Ltd. for planting with a plant spacing of 20 cm and row spacing of 80 cm. Based on the local fertilizer application levels (i.e., normal fertilizer application (NFA) of 600 kg NPK (N: P_2_O_5_: K_2_O = 28: 8: 10) per hectare (ha.); named as 100F), and four fertilizer reduction gradients were set up, i.e., 540 kg/ha (i.e., 90% of NFA; named as 90F), 480 kg/ha (i.e., 80% of NFA; 80F), 375 kg/ha (i.e., 62.5% of NFA; 62.5F), and 300 kg/ha (i.e., 50% of NFA; 50F). Each treatment of fertilizer application was set up with three replicates, and each replicate with an area of 0.1 ha. and randomly distributed in the experimental field (seen in **Figure 1**). Maize was sown on June 16th in 2020, and June 18th in 2021 respectively, and the irrigation and herbicide application were conducted before sowing. After sowing, the experimental field management was completely consistent with the local fields, including no pesticide application, no irrigation, and no topdressing during the entire growing season. On Sept 16th in 2020 and 2021, ten maize plants were randomly selected from each fertilizer treatment replicate to determine the relevant testing indicators, and the rhizosphere soil samples were collected, respectively.

### 2.3 Determination of maize growth indexes

On Sept 16th in 2020 and 2021, ten maize plants were selected from each replicate of fertilizer application treatments to measure plant growth indexes of maize, including plant height (cm), stem diameter (cm), root weight (g) and plant weight (g). The foliar contents of nutrients were determined, including soluble sugars (mg/g), soluble protein (mg/g) and free fatty acids (FFA; mg/g), and the correspondingly used kits were No.A145–1-1, No.A145–1-1 and No.A042–1-1 respectively, which were provided by Nanjing Jiancheng Bioengineering Institute at Nanjing City, Jiangsu Province of China.

On the other hand, the activity of three nitrogen metabolic enzymes [including NADH-GOGAT (nmol/min/g), NR (μmol/h/g), GS (μmol/h/g)] in maize leaves was also determined by using the correspondingly used kits of No BC0075, No BC0080 and No BC0915 respectively, which were provided by Beijing Suolaibao Technology Co. Ltd. at Beijing City of China. And the foliar contents of secondary metabolites, including total phenol (μg/g), tannin (μg/g) and flavonoids (ng/g) were measured by using the colorimetric methods with catechol, gallic acid and rutin as the standard curves (Ali et al., 2022); the hormone contents of JA (pmol/g), SA (pmol/g) and JA-Ile (pmol/g) were further assays were done by using the HPLC-MS/MS (LCMS-8040 system, Shimadzu), referred to Wu et al. (2007).

### 2.4 Measurement of biomass, yield and grain nutrients of maize

On the 9th Oct in 2020, and 10th Oct in 2021, five subplots were randomly selected from each replicated plot of maize field by using the five-point sampling method respectively. From each sampling subplot, 10 ears of maize plants were harvested and dried in an oven for 72 hours at 60°C. The dry weight was then measured by using the same electronic balance (Shanghai Hua Yao Weighing System Co., Ltd.). The following maize yield indexes were measured, including 1000-grain weight (g) and grain yield per ha (kg). The nutrient indexes of the collected maize grains were also determined, including percentage of crude protein (%), and the contents of nitrogen (%), starch (mg/g), total sugars (mg/g), free fatty acids (FFA, nmol/g), and amino acids (μg/g), and the correspondingly used kits were No.PQD-1-G, No.DF-1-Y, No.ZT-1-Y, No.FFAZ-1-W, and No.AA-2-W respectively, which were provided by the Suzhou Keming Biotechnology Co., Ltd at Suzhou City of Jiangsu Province of China.

### 2.5 Insect investigation

From the 4th August to harvest of maize in 2020 and 2021, the insect surveys (including insect pests and natural enemy insects, and the key herbivorous insect of Asian corn borers (*Ostrinia furnacalis*) locally) on maize plants were conducted and counted every ten days to investigate 30 maize plants in each plot of each replicate for the fertilizer application treatments. The community indexes were calculated, including the Shannon-Wiener index (*H*), the Pielou evenness index (*E*), the Margalef richness index (*D*), and the Simpson dominance index (*C*) based on the species and numbers of surveyed insects (Zou et al., 2022). The formulas were as follows:

**(1) *Shannon-Wiener diversity index*:**


$$H=-\sum_{\text{i}=1}^{S}\;P\text{i}\times \ln(P\text{i})\;P\text{i}=N\text{i}/N$$

**(2) *Pielou evenness index*:**


$$E=H/H_{\max}\;H_{\max}=\ln S$$

**(3) *Margalef richness index*:**


D=(S−1)/lnN

**(4) *Simpson dominance index*:**


$$C=\sum_{\text{i}=1}^{S}{\;(P\text{i})}^{2}\;P\text{i}=N\text{i}/N$$

Pi: relative abundance of insect species i; Ni: number of individuals for species i; N: the total number of individuals of all species in the community; *S*: the number of species in the community; *H*_max_: maximum species diversity index.

### 2.6 Soil nitrogen metabolic enzyme activity and composition and diversity of soil microbial community

The soil urease (S-UE) and soil alkaline protease (S-ALPT) were determined by the Suzhou Keming Biotechnology Company’s kits of No.SUE-1-Y and No.SALPT-1-Y respectively, and soil samples were sent to Shanghai Lingen Biotechnology Co., Ltd. for 16S rRNA gene sequencing to analyze soil microbial diversity. The microbial DNA was extracted from a total of 30 soil samples, representing five different nitrogen application treatments with three replicates per treatment over two years of 2020 and 2021. The E.Z.N.A. Soil DNA Kit (Omega Bio-tek, Norcross, GA, USA) was used to extract the DNA according to the manufacturer’s instructions. The V4-V5 region of the bacterial 16S ribosomal RNA gene was amplified by PCR in triplicate, and the pooled DNA product was used to construct an Illumina Pair-End library following standard protocols for genomic DNA library preparation. The amplicon library was paired-end sequenced (2x250) on an Illumina MiSeq platform (Shanghai BIOZERON Co., Ltd.) using standard protocols. Sequence denoising or operational taxonomic units (OTUs) clustering was performed using either the QIIME2 DADA2 analysis process or V-search software analysis process. The relative abundance of soil microbes was determined at the taxonomic level of phylum, and the microbial diversity indices were calculated, including the Chao1 index, Ace index, Shannon index, and Evenness index (Wu et al., 2022; Yao et al., 2022).

### 2.7 Data analysis

The data were analyzed by using the SPSS 20.0, OriginPro 2021 and GraphPad Prism 8.3.0. Two-way ANOVA was used to analyze the effects of sampling year (Y) and fertilizer application (F) on the measured indices of maize and soil microbes, while the two-way repeated-measures ANOVA was used to analyze the impacts of sampling year (Y) and fertilizer application (F), with sampling date as repeated measures, on the indexes of population abundance and community diversity of insects. And the *LSD* test was used to identify the significant differences among treatments at *P*< 0.05.

## 3 Results

### 3.1 Effects of different fertilizer application levels on plant growth of maize

**Figure 2:**
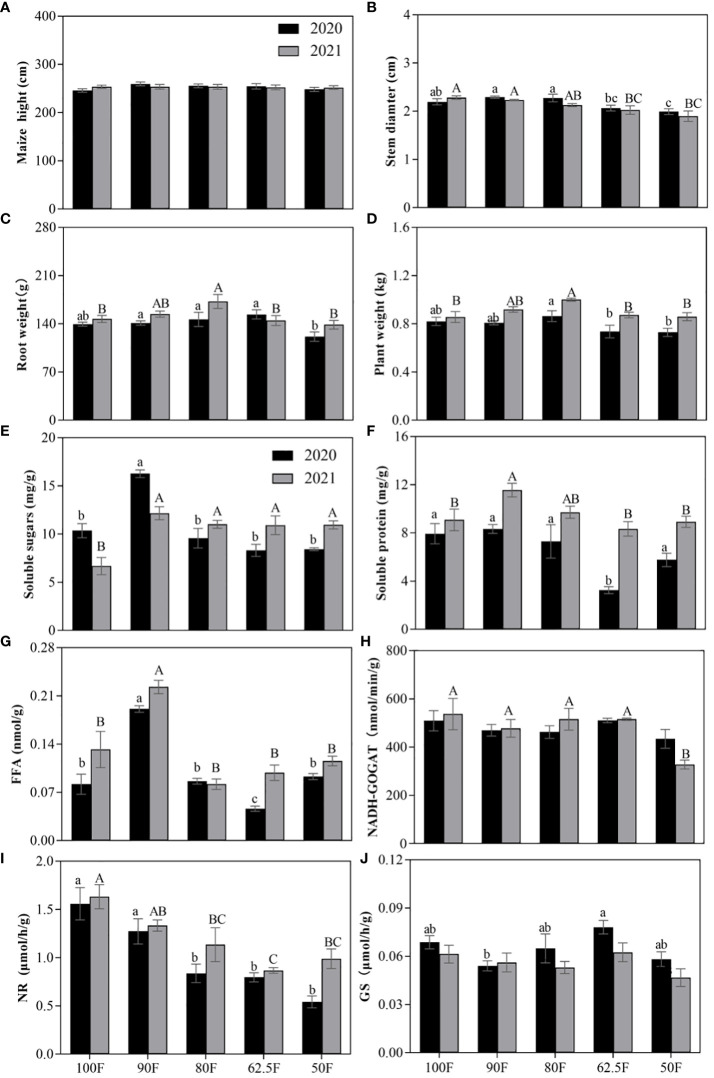
Agronomic traits **(A–D)** of plant growth, foliar nutrition **(E–G)** and nitrogen metabolic enzyme activity **(H–J)** of maize plants during the dough stage under different fertilizer application levels. (Vertical bars denote the standard error of the mean. Different uppercase and lowercase letters represent significant differences among different fertilizer application treatments in 2020 and 2021 respectively, by the *LSD* test at *P*< 0.05. The same as in the following figures).

The plant height of maize at the dough stage, which mainly involves reproductive growth, was not significantly affected by different fertilizer levels, years, or their interaction (*F ≤* 0.91, *P*≥0.46; **Table 1**; **Figure 2A**). However, stem diameter at the dough stage was significantly influenced by different fertilizer application levels (*F=*9.44, *P*<0.001), while sampling year (*F=*1.66, *P*=0.20) and their interaction (*F=*0.94, *P*=0.44) had no significant impact on this index (**Table 1**), and in **Figure 2B** it can be seen that the treatment at 62.5F and 50F significantly reduced the maize stem diameter compared to 100F, respectively. Furthermore, stem diameter declined as fertilizer application level decreased. Root weight and whole plant biomass of maize at the dough stage were significantly affected by both fertilizer levels and sampling year (*F*≥4.98, *P ≤* 0.012), but their interaction did not show a significant effect (*F ≤* 1.85, *P*≥0.13; **Table 1**). The weight of roots and the whole plant increased first and then decreased with decreasing fertilizer application level (**Figures 2C, D**). Based on the experimental results over two years, the maize biomass in the 80F treatment increased by 11.12%, 7.87%, 15.83%, and 17.32% compared to the 100F, 90F, 62.5F, and 50F treatments, respectively. This indicates that the 80F treatment had a significantly positive impact on maize biomass during these two years, resulting in a relatively higher biomass production.

**Table 1:** Two-way ANOVAs for the effects of fertilizer application levels (100F, 90F, 80F, 62.5F, 50F), sampling years (2020, 2021), and their interaction on the agronomic traits of plant growth, foliar nutrition, and nitrogen metabolic enzyme activity of maize during the dough stage (*F*/*P*).

Measured indexes		Fertilizer levels (F)	Years (Y)	F×Y
Agronomic traits	Maize height (cm)	0.91/0.46	0.02/0.90	0.74/0.57
	Stem diameter (cm)	9.44/<0.001^***^	1.66/0.20	0.94/0.44
	Root weight (g)	4.98/0.0011^**^	6.65/0.012^**^	1.85/0.13
	Plant weight (g)	5.12/<0.001^***^	25.62/<0.001^***^	0.78/0.54
Foliar nutrients	Soluble sugars (mg/g)	20.53/<0.001^***^	0.31/0.58	12.15/<0.001^***^
	Soluble protein (mg/g)	9.42/<0.001^***^	44.29/<0.001^***^	2.01/0.13
	Free fatty acids (mg/g)	44.02/<0.001^***^	18.02/<0.001^***^	2.06/0.12
Foliar nitrogen metabolic enzyme activity	NADH-GOGAT (nmol/min/g)	5.09/0.005^**^	0.01/0.91	1.50/0.24
	NR (μmol/h/g)	19.85/<0.001^***^	7.40/0.013^*^	1.25/0.32
	GS (μmol/h/g)	3.66/0.022^*^	6.78/0.017^*^	0.80/0.54

100F, 90F, 80F, 62.5F, 50F – 100%, 90%, 80%, 62.5% and 50% of the normal fertilizer application (NFA) of 600 kg NPK (N: P_2_O_5_: K_2_O = 28: 8: 10) per hectare (ha.), respectively; ^*^P<0.05; ^**^P<0.01; ^***^P<0.001.

The nutrient level and the activities of nitrogen metabolic enzymes in maize leaves reflect the ability of maize to metabolize and accumulate nutrients. Fertilizer application level significantly affected the contents of three soluble nutrients in maize leaves (*F*≥9.42, *P*<0.001; **Table 1**). Sampling year had no significant effect on foliar content of soluble sugar (*F=*0.31, *P*=0.58), but the interaction of fertilizer levels and sampling year had a significant effect on soluble sugar in leaves (*F*=12.15, *P*<0.001; **Table 1**). And sampling year had significant effects on the contents of soluble protein (*F*=42.29, *P*<0.001) and free fatty acid content (*F*=18.02, *P*<0.001) in maize (**Table 1**). From the **Figures 2E–G** showed that with the reduction of fertilizer application level, the foliar contents of soluble nutrients first increased and then decreased, showing the highest level at 90F.

Fertilizer application level had significant effects on the activities of nitrogen metabolic enzymes in leaves (*F*≥3.66, *P ≤* 0.022; **Table 1**), and sampling year had a significant effect on the nitrogen metabolic enzymes of GS and NR activity in leaves (*F*≥6.78, *P ≤* 0.017; **Table 1**). The enzyme activity of NADH-GOGAT in the maize leaves of 50F was significantly lower than that of other treatments in 2021 (*P*<0.05; **Figure 2H**), while the enzyme activity of GS in leaves showed no significant difference among different fertilizer application treatments in 2021, with the lowest at 80F and the highest at 62.5F, and the treatment of 90F and 62.5F had a significant difference in 2020 (**Figure 2J**). The activity of NR was highest at 100F and decreased with the decrease in fertilizer application (**Figure 2I**).

### 3.2 Effects of different fertilizer application on secondary metabolites and hormones in maize leaves

**Figure 3:**
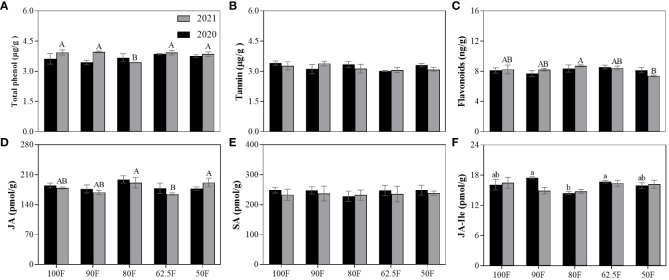
Secondary metabolites **(A–C)** and plant hormones **(D–F)** in maize leaves during the dough stage under different fertilizer application treatments. (Vertical bars denote the standard error of the mean. Different uppercase and lowercase letters represent significant differences among different fertilizer application treatments in 2020 and 2021 respectively, by the *LSD* test at *P*< 0.05. The same as in the following figures).

The metabolites and hormones of maize are related to maize growth and insect resistance. Reduced fertilizer application only had a significant effect on JA (*P*<0.05; **Table 2**). **Figure 3** shows that the total phenol content of 80F in 2021 was significantly lower than other treatments, while tannin content showed no significant difference between treatments. The lowest flavonoid content level was observed for 50F (**Figure 3C**). JA content was highest for 80F, lowest for 62.5F. Combining the results from two years of experiments, the JA content in the 80F treatment was 5.78%-13.9% higher than that in the other treatments. Additionally, the lowest level of JA-Ile is found in the treatment of 80F, which decreased by 9.07%-11.7% compared to that in the other treatments (**Figures 3D, F**).

**Table 2:** Two-way ANOVAs of fertilizer levels (100F, 90F, 80F, 62.5F, 50F), sampling years (2020, 2021) and their interaction on the secondary metabolites and plant hormones of maize plants during the dough stage (*F*/*P*).

Measured indexes		Fertilizer level (F)	Years (Y)	F×Y
Secondary metabolites	Total phenol (μg/g)	1.87/0.16	3.42/0.079	2.38/0.086
	Tannin (μg/g)	1.04/0.41	0.25/0.62	0.97/0.45
	Flavonoids (ng/g)	1.98/0.14	0.01/0.82	1.04/0.41
Plant hormone	JA (pmol/g)	2.89/0.048^*^	0.65/0.43	0.86/0.50
	SA (pmol/g)	0.61/0.66	1.79/0.20	0.27/0.89
	JA-Ile (pmol/g)	2.70/0.063	0.80/0.38	1.82/0.17

^*^P<0.05.

### 3.3 Effects of different fertilizer application on grain yield and nutrients of maize

**Figure 4:**
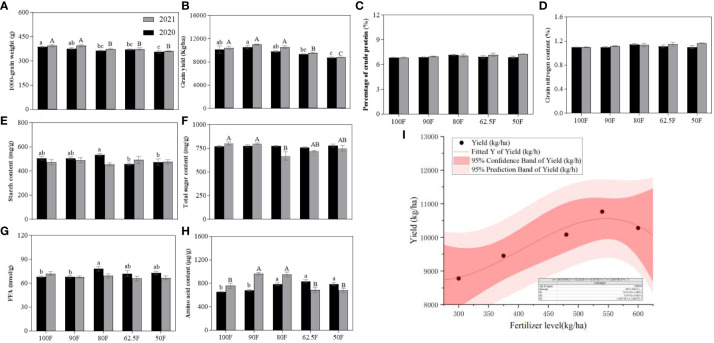
Grain yield **(A, B)** and nutrients **(C–H)** of maize with different fertilizer application treatments in 2020 and 2021; and Fitting function **(I)** based on the relationship between maize yield and fertilizer application (300, 375, 480, 540 and 600 kg/ha) of NPK (N: P_2_O_5_: K_2_O = 28: 8: 10) in 2020 and 2021.

Yield and nutritional level of maize grain are the main objectives of planting. Fertilizer levels and sampling years had significant effects on maize 1000-grain weight (g) and grain yield (kg/ha) (*P*<0.041; **Table 3**), but the interaction between fertilizer levels and sampling years had no significant effect on it. For 1000 grain weight and grain yield, there was a tendency to decrease with fertilizer reduction (**Figures 4A, B**). Based on the two-year experimental results, the maize grain yield in the 90F treatment increased by 4.72%, 5.73%, 13.87%, and 22.63% compared to that of the treatments of 100F, 80F, 62.5F, and 50F treatments, respectively.

**Table 3:** Two-way ANOVAs of fertilizer levels (100F, 90F, 80F, 62.5F, 50F), sampling years (2020, 2021) and their interaction on the grain yield and nutrients of maize (*F*/*P*).

Measured indexes		Fertilizer levels (F)	Years (Y)	F×Y
Maize yield	1000-grain weight (g)	11.75/<0.001^***^	7.43/0.013^*^	0.56/0.70
	Grain yield (kg/ha)	18.17/<0.001^***^	4.77/0.041^*^	0.53/0.71
Grain nutrients	Percentage of crude protein (%)	1.95/0.14	3.30/0.08	1.22/0.33
	Grain nitrogen content (%)	1.95/0.14	3.30/0.08	1.22/0.33
	Starch content (mg/g)	0.75/0.57	2.40/0.14	2.62/0.066
	Total sugar content (mg/g)	4.33/0.011^*^	3.01/0.10	3.96/0.016^*^
	FFA (nmol/g)	1.63/0.21	5.37/0.031^*^	2.31/0.093
	Amino acid content (μg/g)	9.61/<0.001^***^	10.03/0.005^**^	18.80/<0.001^***^

^*^P<0.05; ^**^P<0.01; ^***^P<0.001.

There was no significant difference in crude protein percentage (%), grain nitrogen content (%), or starch content between fertilizer treatment and year (**Table 3**; **Figures 4C, D**). In 2020, starch content showed that 62.5F and 50F were significantly lower than 80F (*P*<0.05; **Figure 4E**). Fertilizer treatment had a significant effect on total sugar content, while the year had no significant effect. The interaction between the two had a significant effect, but the FFA content was the opposite (**Table 3**). The fertilizer levels, years, and their interaction had significant effects on the amino acid content (*P*<0.005; **Table 3**). In 2021, the total sugar content of 80F was significantly lower than that of 100F and 90F, but there was no significant difference in contrast with others (**Figure 4F**). While in 2020, the FFA content of 80F was significantly higher than that of 100F and 90F, but there was no significant difference in other fertilizer levels (**Figure 4G**). Amino acid content showed a large difference, and combined with two years of results, amino acid content is at a higher level at 80F treatment (**Figure 4H**). According to the yield results of two years and fitting curve analysis, the fertilizer level with the highest expected yield (i.e., 10558.0 kg/ha) was 546.9 kg/ha (**Figure 4I**).

### 3.4 Effects of different fertilizer application on the community indexes of insects on maize plants

**Figure 5:**
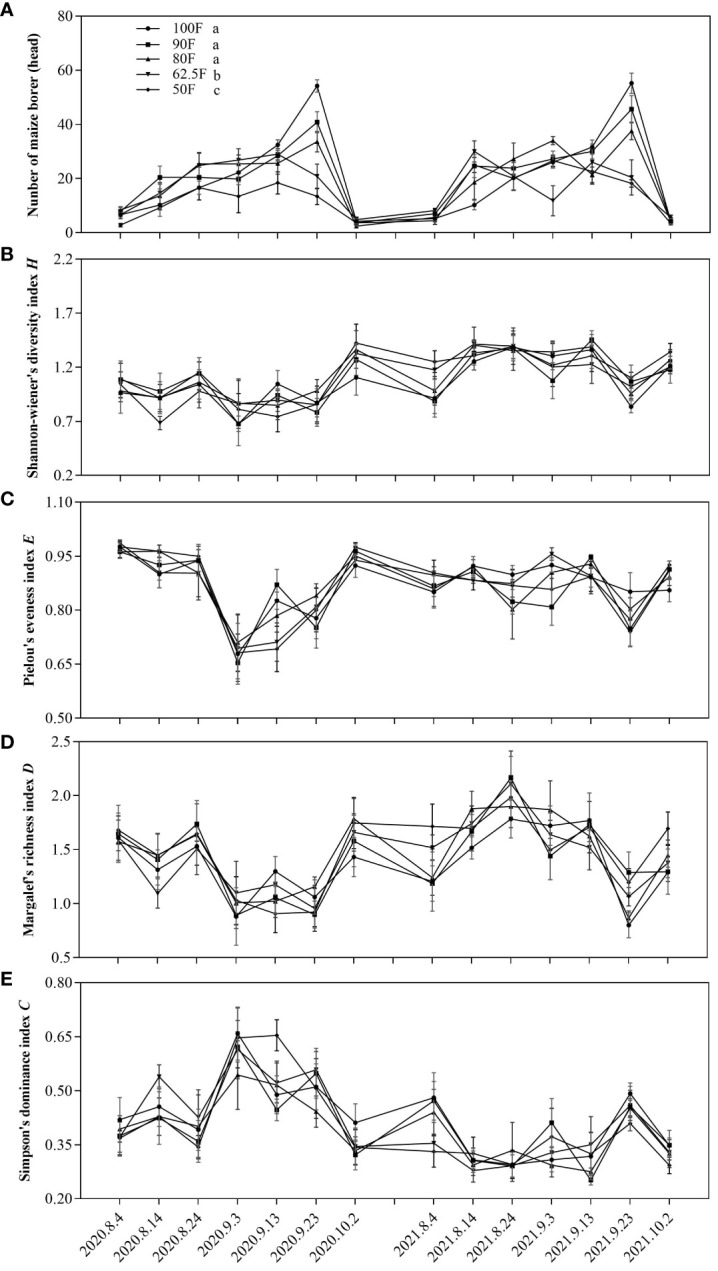
Dynamics of the population dynamics of key herbivorous insect, Asian corn borers (*Ostrinia furnacalis*) **(A)**, and community indexes of insects on maize plants **(B–E)** under different fertilizer application treatments in 2020 and 2021.

The levels of insect diversity in the field was not significantly different among the different treatments, while the years had a significant effect on the Shannon-Wiener index (*H*), Margalef richness index (*D*), and dominance index (*C*). Fertilizer level (*F*=15.94, *P*<0.001), sampling year (*F*=10.83, *P*=0.0023) and their interaction (*F*=3.16, *P*=0.024) have significant effect on the occurrence of Asian corn borers (**Table 4**). Compared with the normal fertilizer level (100F), 62.5F and 50F both significantly reduced the occurrence of Asian corn borers (*P*<0.05; **Figure 5**). And the occurrence of Asian corn borers for the treatment of 50F was significantly lower than that for the treatment of 62.5F (*P*<0.05; **Figure 5**).

**Table 4:** Two-way repeated-measures ANOVAs of fertilizer levels (100F, 90F, 80F, 62.5F, 50F), sampling years (2020, 2021) and their interaction (with sampling dates as repeated measures) on the community indexes of insects on maize plants (*F*/*P*).

Measured indexes	Fertilizer level (F)	Years (Y)	F×Y
Asian corn borers (individuals/100 plants)	15.94/<0.001^***^	10.83/0.0021^**^	3.16/0.024^*^
Shannon-Wiener index (*H*)	0.63/0.64	57.30/<0.001^***^	0.17/0.95
Pielou evenness index (*E*)	0.29/0.89	1.83/0.18	0.77/0.55
Margalef richness index (*D*)	0.81/0.53	25.26/<0.001^***^	0.48/0.75
dominance index (*C*)	0.64/0.64	81.335/<0.001^***^	0.89/0.48

^*^P<0.05; ^**^P<0.01; ^***^P<0.001.

Fertilizer level, and its interaction with sampling year haven’t significant effects on the community indexes of insects (*P*>0.05; **Table 4**). While there were significant differences in Shannon-Wiener index (*H*), Margalef richness index (*D*) and dominance index (*C*) of insects among sampling years (*P*<0.001; **Table 4**).

### 3.5 Effects of different fertilizer application on the enzyme activity of soil nitrogen metabolism and composition and diversity of soil microbial community in maize field

**Figure 6:**
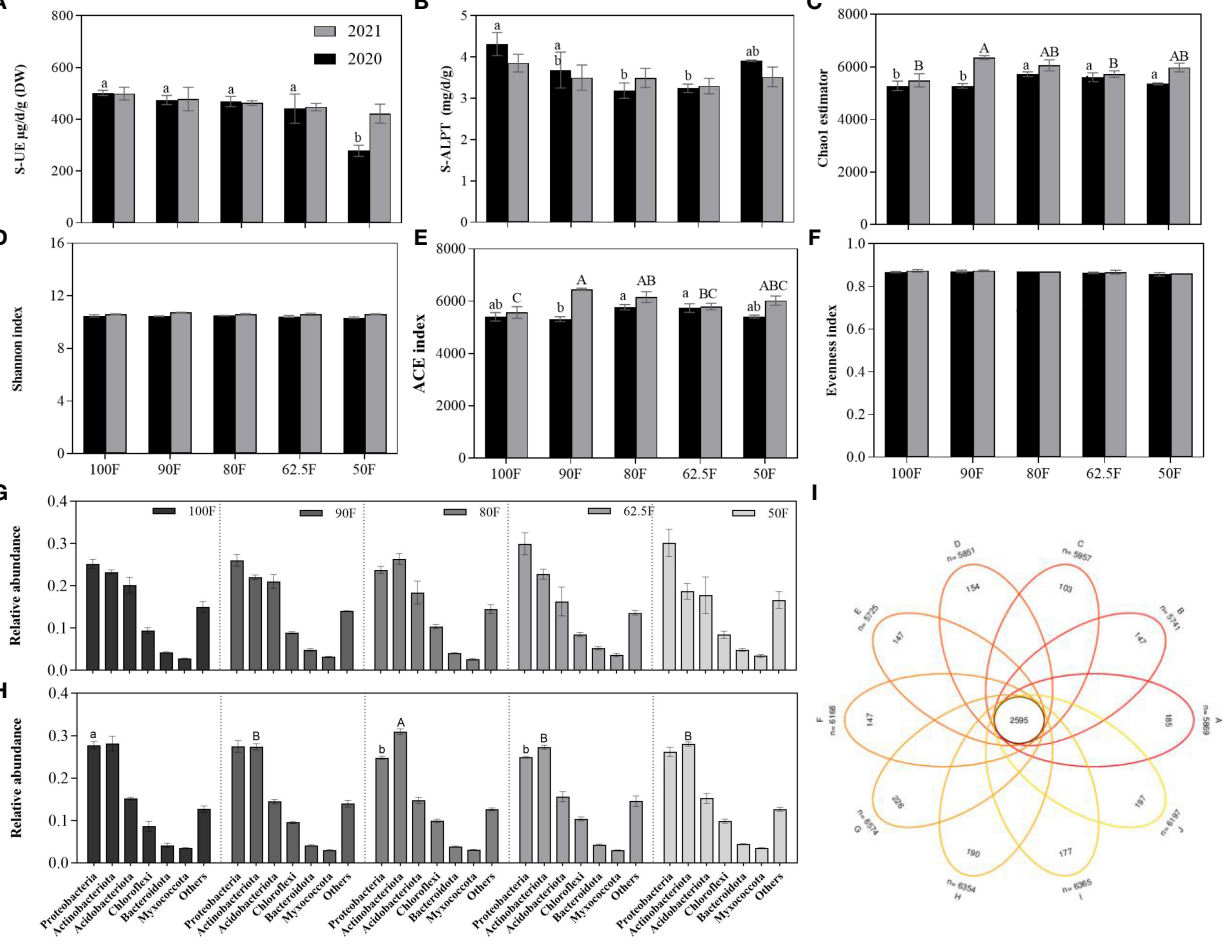
Enzyme activity of soil nitrogen metabolism **(A, B)** and community indexes of soil microbes in maize field **(C–F)**; The relative abundances of soil microbes at different phylum levels [**(G)**2020 and **(H)** 2021], and the Venn diagrams of soil microbes **(I)** in maize field under different fertilizer application treatments. (Different elliptical circles represented different samples in **(I)**, and the numbers in the center and edges represent the common and unique OTUs for the five fertilizer application treatments of 100F, 90F, 80F, 62.5F and 50F. 2020: **(A–E)**; 2021: **(F–J)**. respectively.). (Vertical bars denote the standard error of the mean. Different uppercase and lowercase letters represent significant differences among different fertilizer application treatments in 2020 and 2021 respectively, by the *LSD* test at *P*< 0.05. The same as in the following figures).

Fertilizer application can directly affect the composition of microorganisms in the soil, which in turn affects the nitrogen metabolizing enzyme activities of the soil. Fertilizer level had significant impacts on soil nitrogen metabolic enzyme activities of S-UE (*F*=7.76, *P*=0.0041) and S-ALPT (*F*=3.55, *P*=0.024), while sampling year and its interaction with fertilizer level had no significant effects on the enzyme activities of S-UE and S-ALPT (*P*>0.13; **Table 5**). Compared with the normal fertilizer treatment (100F), just 50F significantly reduced the enzyme activity of S-UE in soil in 2020 (*P*<0.05; **Figure 6A**), and 80F and 62.5F both significantly decreased the enzyme activity of S-ALPT in soil in 2020 (*P*<0.05; **Figure 6B**).

**Table 5:** Two-way ANOVAs of fertilizer levels (100F, 90F, 80F, 62.5F, 50F), sampling years (2020, 2021) and their interaction on the enzyme activity of soil nitrogen metabolism and soil microbial diversity in maize field (*F*/*P*).

Measured indexes		Fertilizer levels (F)	Years (Y)	F×Y
Enzyme activity of soil nitrogen metabolism	S-UE μg/d/g (DW)	7.76/0.0041^**^	2.40/0.15	2.35/0.13
	S-ALPT (mg/d/g)	3.55/0.024^*^	0.76/0.39	0.85/0.51
Soil microbial diversity	Chao1 index	3.20/0.035^*^	23.84/<0.001^***^	3.15/0.037^*^
	Shannon index	1.32/0.30	28.55/<0.001^***^	0.99/0.43
	Ace index	3.13/0.041^*^	25.91/<0.001^***^	4.28/0.012^*^
	Evenness index	1.76/0.18	3.31/0.082	0.47/0.76

^*^P<0.05; ^**^P<0.01; ^***^P<0.001.

The Chao1 index and ace index at different fertilizer levels, years, and their interaction were significantly different (*F*≥3.13, *P ≤* 0.041). For the Shannon index, only the Sampling year had a significant impact (*F*=28.55, *P*<0.001). The Chao1 and ace indices increased first and then decreased with the decreasing fertilizer levels, reaching their highest levels at 90F and 80F (**Figures 6C, E**).

The number of soil microbial OTUs can be as the supplement information diversity index (**Figure 6I**). As far as the Soil microbes’ relative abundances are concerned, the change in relative abundances brought by reduced application of fertilizers is also small. Only in 2021, compared with treatment 100F, 80F and 62.5F significantly reduced the relative abundance of Proteobacteria, and the relative abundance of Actinobacteriota with treatment 80F was significantly higher than that of treatment 90F, 62.5F and 50F (**Figure 6H**).

### 3.6 Correlation analysis of maize yield and other testing index under different fertilizer application conditions

**Figure 7:**
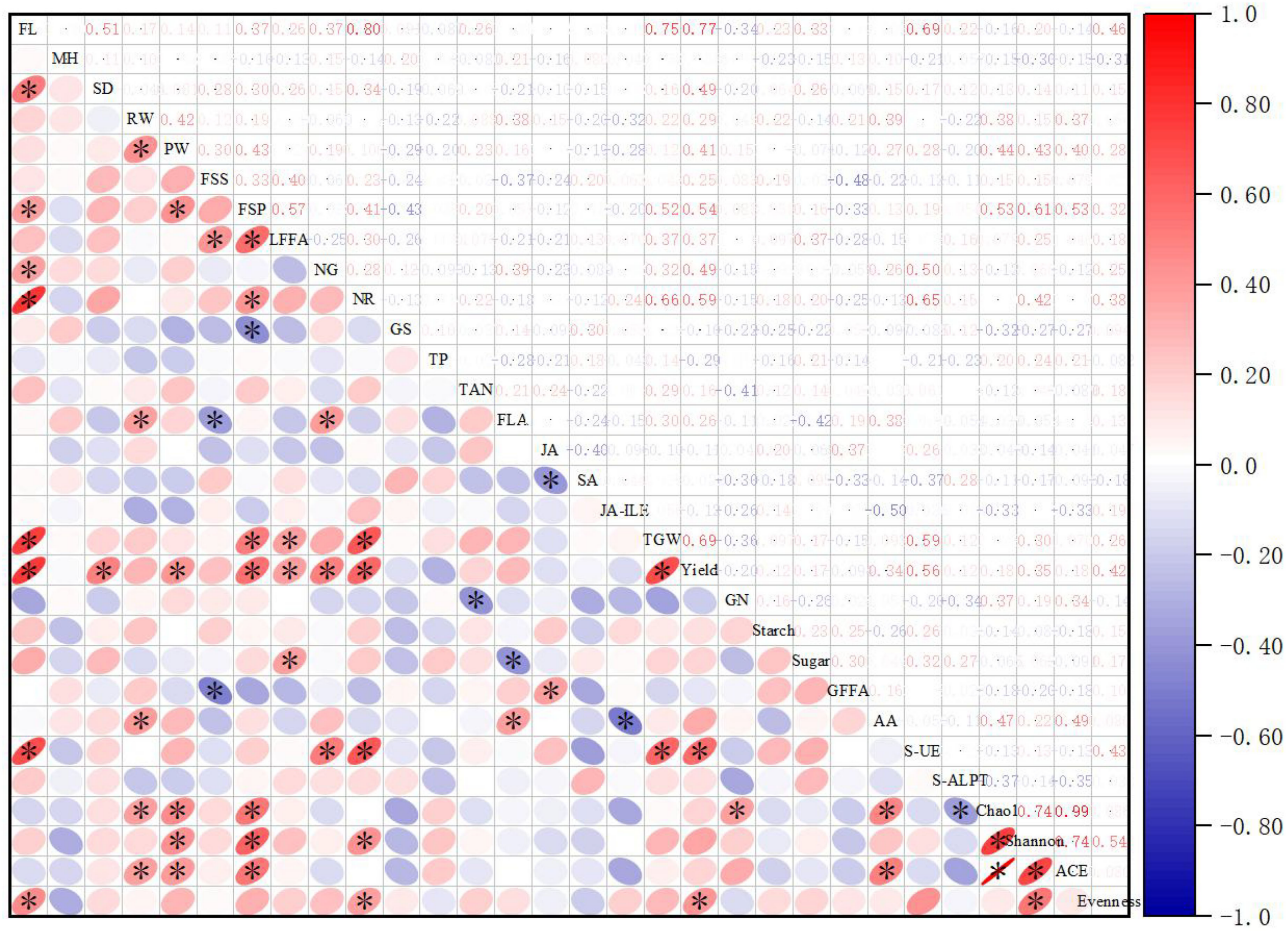
Correlation analysis. ^*^ *p<* 0.05; FL, fertilizer level; MH, maize height; SD, stem diameter; RW, root weight; PW, plant weight; FSS, foliar soluble sugars; FSP, foliar soluble protein; LFFA, leaf free fatty acids, NG, glutamine oxoglutarate aminotransferase, NR, nitrate reductase; GS, glutamate synthetase; TP, total phenol; TAN, tannin; FLA, flavonoids; JA, jasmonic acid; SA, salicylic acid; JA-ILE-jasmonoyl-L-isoleucine; TGW, 1000-grain weight; Yield, grain yield; GN, grain nitrogen content; Starch, grain starch; Sugar, grain sugar; GFFA, grain free fatty acids; AA, grain amino acid content; S-UE, soil urease; S-ALPT, soil alkaline protease).

The main focus in the correlation analysis was on the relationship between fertilizer application and maize yield, and as shown in **Figure 7**, fertilizer level (at 300–600 kg/ha) showed a significant positive correlation with SD, FSP, NG, NR, TGW, Yield, S-UE, Evenness. And Yield had a significant positive correlation with FL, SD, PW, FSP, LFFA, NG, NR, S-UE, and Evenness.

## 4 Discussion

### 4.1 Effect of reduced fertilizer application on growth index of maize

In recent years, there has been a growing awareness of the need for sustainable production practices in agriculture, particularly in terms of fertilizer use (Feyissa et al., 2022; Liu et al., 2022; Tian et al., 2022). While fertilizers have a strong positive correlation with maize yield, the studies have shown that fertilizer application in China far exceeds actual crop requirements (Wang et al., 2022c). The pursuit of both high yields and sustainable production has become a dual demand on the land (Wang et al., 2022a).

This field experiment was conducted to investigate the impact of reduced fertilizer application on maize growth and yield. In this study, the highest application rate of 600 kg/ha of compound fertilizer, the reduced application of fertilizer had a significant impact on the corn stem diameter, root weight, plant weight, yield, soluble sugars, soluble protein, free fatty acids, NADH-GOGAT, NR, GS, had a significant impact, and in the correlation analysis, stem diameter, foliar soluble protein, glutamine oxoglutarate aminotransferase, nitrate reductase, nitrate reductase, 1000-grain weight, yield, soil urease, and evenness index of soil microbial diversity, had a significant positive correlation with it. Among the tested indexes, 90F and 80F are mostly at a high level, but this difference is often not significant. Fertilizer level has a significant effect on maize yield index, 1000-grain weight and grain yield, but it only had a significant effect on total sugar and amino acids in the determine of grains nutrition, just like agronomic indexes, which show higher values at 90F and 80F.

Through longer time and monitoring of more indicators, we can think that the current reduction of fertilizer application by 10% to 20% will not cause yield reduction. The fitting curve between fertilizer and yield, on the other hand, provides evidence that the fertilizer application amount of 547 kg/ha is optimum, while the minimum fertilizer application amount of 486 kg/ha will not reduce the yield compared with the present. Reducing the application of fertilizer by 10% not only did not bring about a reduction in yield but was beneficial to the increase in corn yield. In addition, the content of soluble nutrients was significantly increased and the enzyme activity of nitrogen metabolism was not significantly decreased by reducing the application of fertilizer by 10%. The decrease in fertilizer application had no obvious effect on maize biomass. When the field fertilizer is reduced by 50%, the growth of corn is obviously adversely affected.

Field test models demonstrated that reducing a certain amount of nitrogen combined with appropriate fertilization mode was not affected crop yield, but rather reduced eluviations and N_2_O emissions and environmental footprints (Shao et al., 2016; Banger et al., 2020; Huang et al., 2021; Wang et al., 2022b). The nutrients and nitrogen metabolism of plant leaves reflect the growth of crops when the fertilizer level was reduced. Studies have shown that a higher degree of fertilizer reduction will reduce the nutrients in corn leaves and reduce crop yield (Li et al., 2022a; Li et al., 2022b). Combined with our work, we can think that it is possible and worthwhile to lose weight properly in the local area.

A coupling analysis of crop yield, nitrogen input and farmers’ technical transformation efficiency in 241 representative areas of winter wheat and summer maize production in the north China Plain showed that only 15% of the winter wheat and 4% of the summer maize in the farmland achieved the synergistic effects of high yield and high-efficiency fertilization, and the farmers’ technical transformation efficiency was relatively low (Li et al., 2019). The use of linear and logistic regression models has demonstrated that the continued excessive use of fertilizer in maize cultivation in northern China despite the recent decline in fertilizer application, and the bigger the farm is, the less chemical fertilizer is invested and the higher the output is (Yu et al., 2023). This phenomenon indicates that family farmers who engage in intensive agriculture lack technical guidance and high-yield conditions, ultimately investing more resources without reaping the desired level of returns. And the year and region will have an obvious influence on the optimum fertilizer consumption of maize (Dhital and Raun, 2016).The purpose of our experiment is more inclined to provide suggestions on fertilizer application for local farmers’ production in recent years. The current results show that reducing fertilizer application by 20% will not affect production, but this does not mean that such fertilizer input is the most suitable. Past research showed that the fertilizer utilization efficiency in North China Plain is low (Liu et al., 2020; Hu et al., 2023). If a fertilizer more suitable for local soil is produced and used, a lower fertilizer consumption will be possible (Li et al., 2021; Feyissa et al., 2022).

### 4.2 Effects of reduced fertilizer application on secondary metabolites and hormones in maize leaves and the insect occurrence in the field

Studies have shown that higher fertilizer inputs promote the occurrence of pests, which in turn can be detrimental to production (Jiang and Schulthess, 2005; Rashid et al., 2017; Song et al., 2020; Wang et al., 2022a). Total phenols, tannins, and flavonoids are important for resistance of maize, including resistance to pests, diseases, and extreme environments (Wang et al., 2022a). However, in our experiment, the change of fertilizer level was not lead to significant changes in the content of these substances in corn leaves. The analysis of variance of repeated measurements for two years showed that the reduction of fertilizer application had no significant effect on the insect diversity index in the field, while the reduction of fertilizer application (62.5F and 50F) reduced the occurrence of mainly pest Asian corn borer. This work further emphasizes the need to correct the excessive use of fertilizer.

JA, SA, and JA-ILE belong to plant hormones that play a critical role in defense signal transduction (Eitle et al., 2019). Adverse conditions can significantly affect the production of these substances, which then play their anti-stress roles, such as pest and disease resistance (Schmelz et al., 2003; Couture et al., 2010; Batool et al., 2022). They also regulate stress resistance, including insect resistance in maize (Wang et al., 2021). However, we found no significant effect of fertilizer levels on SA and JA-Ile content. The levels of JA content are shown separately in **Figure 3D**, and there were no significant differences between 100F, 90F, and 80F. JA and SA are secondary metabolites that increase after damage, but the levels of JA and SA in different treatments were similar, and indicating that the difference in insect damage levels among the different treatments was small. This was in line with the outcome of the survey on insect diversity.

Although the use of fertilizers, particularly nitrogen, has advantages for reducing plant stress and enhancing insect resistance, insects exhibit a tendency to target plants with higher nutrient levels (Wang et al., 2022a). In our experiment, it was found that the main local corn pest was Asian corn borer, which significantly reduced its occurrence in treatments 62.5F and 50F, while the difference among other treatments was not significant. The change of fertilizer application rate did not significantly affect the diversity of insects in the field, indicating that it had little impact on the ecology of farmland.

Based on the field insect survey and the growth of maize, we concluded that low-level fertilizer reduction (10%-20%) had no significant effect on the insect resistance index of corn or the occurrence of insects in the field. Rigorous pot experiments have proved that reduced fertilizer application can increase the content of crop JA and other resistant substances (Song et al., 2020), but we did not find significant changes in the field experiments. We speculated that, this was because the crops in the North China Plain were cropped two times a year, the wheat straw was pulverized and returned to the field, and the wheat fertilizer residue and straw still provide N, P, K, and others, and studies supported this speculation which makes the impact of fertilizer reduction on corn even lower (Liu et al., 2016; Luo et al., 2022; Pu et al., 2022).

### 4.3 Soil nitrogen metabolic enzyme activity and composition and diversity of soil microbial community

Studies have shown that reducing fertilizer input can prevent soil acidification and salinization, and increase the abundance of soil bacteria, but it can also reduce soil digestion and denitrification (Sun et al., 2022). Further, Nitrate nitrogen is soluble in water and is therefore easily lost through farmland runoff and porous soil. Nitrogen can also be lost from waterlogged soil to the atmosphere by denitrification (Kitchen et al., 2022).

S-UE and S-ALPT were indicators of the activity of two soil nitrogen metabolic enzymes, reflects the nitrogen metabolism ability of soil microorganisms (Akhtar et al., 2019). In our study, we found that when the fertilizer was reduced by 0–37.5%, the activity of S-ALPT was reduced, but there was no significant effect on S-UE. In the experiment in 2020, the enzyme activity of S-ALPT was significantly reduced by treatments with reduced fertilization amounts of 80F and 62.5F, but there was no significant difference in 2021., and an appropriate reduction in fertilizer application was conducive to improving soil microbial diversity in this study (**Figure 6**). Higher soil nutrients and bacterial diversity played a significant role in maintaining soil fertility and the balance of soil microbial communities (Muhammad et al., 2022). The application of fertilizer and the growth of corn are conducive to the metabolic activities of soil microorganisms, which in turn act on the growth of maize (Rao et al., 2021). In agro-ecosystems with high nitrogen input, moderately reducing subsequent nitrogen supply should be the priority choice for soil management (Sarula et al., 2022).

## 5 Conclusion

**Figure 8:**
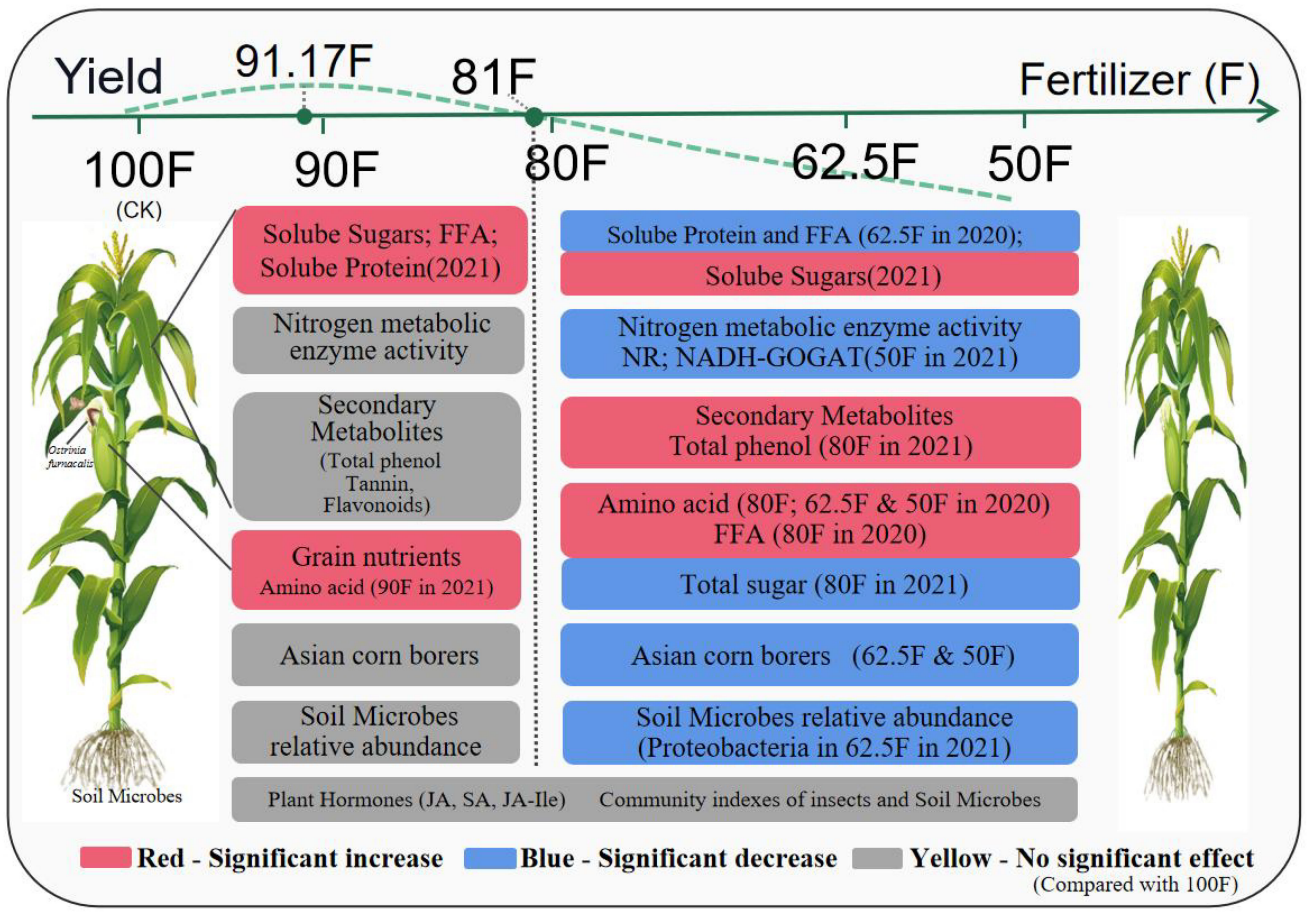
Effects of reduced fertilizer application on plant growth and yield of maize crop, and population abundance of key insect pests and community diversity of insects and soil microbes. (Fertilizer - 100% (Control, 100F), 90% (90F), 80% (80F), 62.5% (62.5F) and 50% (50F) NFA (normal fertilizer application) of 600 kg NPK (N: P_2_O_5_: K_2_O = 28: 8: 10) per hectare (ha.); FFA, Free fatty acids; NR, Nitrate reductase; NADH-GOGAT, NADH-dependent glutamate synthase; JA, Jasmonic acid; SA, Salicylic acid; JA-Ile, Jasmonoyl-isoleucine).

A two-year fertilizer reduction experiment was conducted based on local agricultural production practices. From the assessment of maize growth, field insect occurrence, and soil microbial analysis regarding the feasibility of reducing fertilizer, it is determined that the optimal fertilizer application rate is approximately 547kg/ha. However, a reduction to 486 kg/ha does not result in decreased yield. This sort of experimental evidence is helpful to guide the local actual production, further, it gives additional information for the farmer to undergo to minimize the fertilizer use. Because as far as the experimental results are conservative, reducing the use of fertilizers by 10%-20% will not cause a reduction in production (**Figure 8**). Based on the earlier report, locations, and years showed extensive variation in the optimal fertilizer application for maize, so this work gives further concrete information for maize production in Jinan only for a short time.

## Data availability statement

The raw data supporting the conclusions of this article will be made available by the authors, without undue reservation.

## Ethics statement

The manuscript presents research on animals that do not require ethical approval for their study.

## Author contributions

YaZ: Investigation, Writing – original draft, Formal analysis. LL: Investigation, Data curation, Writing – review & editing. YW: Data curation, Investigation, Writing – review & editing. RD: Data curation, Investigation, Writing – review & editing. HD: Data curation, Investigation, Writing – review & editing. YuZ: Data curation, Investigation, Writing – review & editing. ZD: Data curation, Investigation, Writing – review & editing. FC: Conceptualization, Funding acquisition, Supervision, Writing – review & editing.
